# A Reconfigurable Surface-Plasmon-Based Filter/Sensor Using D-Shaped Photonic Crystal Fiber

**DOI:** 10.3390/mi13060917

**Published:** 2022-06-09

**Authors:** S. Selvendran, J. Divya, A. Sivanantha Raja, A. Sivasubramanian, Srikanth Itapu

**Affiliations:** 1 School of Electronics Engineering (SENSE), Vellore Institute of Technology, Chennai 600127, Tamil Nadu, India; divyaselva0605@gmail.com (J.D.); sivasubramanian.a@vit.ac.in (A.S.); 2 Department of ECE, Alagappa Chettiar Government College of Engineering and Technology, Karaikudi 630003, Tamil Nadu, India; sivanantharaja@yahoo.com; 3 Department of ECE, Alliance College of Engineering and Design, Alliance University, Bengaluru 562106, Karnataka, India; srikanth.itapu@alliance.edu.in

**Keywords:** polarization filter, photonic crystal fiber, surface plasmon resonance, plasmonic sensor, silver

## Abstract

A reconfigurable surface-plasmon-based filter/sensor using D-shaped photonic crystal fiber is proposed. Initially a D-shaped PCF is designed and optimized to realize the highly birefringence and by ensuring the single polarization filter. A tiny layer of silver is placed on the flat surface of the D-shaped fiber with a small half-circular opening to activate the plasmon modes. By the surface plasmon effect a maximum confinement loss of about 713 dB/cm is realized at the operating wavelength of 1.98 µm in X-polarized mode. At this wavelength the proposed fiber only allows Y-polarization and filters the X-polarization using surface plasmon resonance. It is also exhibiting maximum confinement loss of about 426 dB/cm at wavelength 1.92 µm wavelength for Y-polarization. At this 1.92 µm wavelength the proposed structure attenuated the Y-polarization completely and allowed X-polarization alone. The proposed PCF polarization filter can be extended as a sensor by adding an analyte outside this filter structure. The proposed sensor can detect even a small refractive index (RI) variation of analytes ranging from 1.34–1.37. This sensor provides the maximum sensitivity of about 5000 nm/RIU; it enables this sensor to be ideally suited for various biosensing and industrial applications.

## 1. Introduction

Photonic crystal fibers (PCF) are a new type of fiber that has a flexible structure and various unique features, such as high nonlinearity [1], engineered dispersion profile over the region of interest [2,3], high birefringence [4], endless single mode, low transmission loss [2,3,4,5] and easy filling of materials in fiber [6,7,8]. PCF has been extended to plasmonic devices by filling them with various metals, such as gold [9,10,11,12,13], silver [14,15], copper [16] and titanium [17]. Surface plasmon (SP) develops at the metal/dielectric interface when plasmonic materials are exposed to electromagnetic waves due to the collective oscillations of conduction electrons [18]. At the phase-matching condition, the core-guided mode transfers maximum energy to the plasmon mode. As a result of coupling, core mode energy decreases, and confinement loss increases. This phenomenon is known as surface plasmon resonance (SPR) [19].

PCF-based plasmonic devices have gained popularity because of their small size, versatility and controllability. Sensors [20], polarization filters [21], multiplexers–demultiplexers [22] and polarization splitters [23] are few examples of PCF-based plasmonic devices applications. In communication systems, a polarization filter is an essential element. Metal-coated or metal-filled plasmonic polarization filters have gained a lot of interest in recent years. When the core-guided mode is phase matched with the plasmon mode at a specific wavelength, SPR occurs. It will also absorb the power of one polarized mode while allowing the other to pass through. As a result, a PCF-based polarization filter can filter different polarizations. The SPR is present in only a few metals; these noble metals, such as gold and silver, are widely investigated in SPR due to their high stability.

In recent years, many researchers have proposed novel PCF-based polarization filters. In 2017, Ying et al. reported a silver-filled tunable single-polarization filter. Their reported confinement losses for X-polarization were 371 dB/cm and 252 dB/cm at the operating wavelengths of 1310 nm and 1550 nm, respectively, whereas Y-polarization mode exhibited very low confinement losses of about 14 dB/cm and 10 dB/cm, respectively. For a propagation distance of 1 mm, a bandwidth of 179 and 71 nm was obtained at 1310 and 1550 nm wavelengths [24]. Manish Sharma, et al. (2019) reported an RI sensor using an annular core photonic crystal fiber. This work exhibits high sensitivity over a large dynamic range. In particular, in the RI range from 1.31 to 1.39, this proposed fiber displays more propagation loss to sense the biochemicals, and its recorded sensitivity is 2.65 × 10^4^ (dB/m)/RIU at 1550 nm and 7.83 × 10^3^ (dB/m/) RIU at 980 nm [25]. Xue et al. published a paper in 2018 that described a novel offset core filled with a gold layer photonic crystal fiber filter. At a wavelength of 1.55 µm, the confinement loss of a Y-polarized light was 657 dB/cm, while the X-polarized light was comparatively low. At a wavelength of 1.55 µm, the cross-talk of 56.2 dB with a bandwidth of 100 nm was obtained for a fiber length of 1 µm [26]. In that year, Yang et al. developed a high-birefringence PCF filter with silver layers that was selectively coated. At a 1.31 µm operating wavelength, a confinement loss of 500 dB/cm was obtained, and a full-width half-maximum was 23 nm [27]. Liu et al. suggested a single-polarization bimetal-coated (Au/Ag) PCF filter with liquid-filled air holes in 2019. Confinement losses for the Y-polarized mode were 544.3 dB/cm and 147.3 dB/cm for a wavelength of 1.31 µm and 1.55 µm, respectively. In the same wavelength, the X-polarized losses were 12.3 dB/cm and 24.0 dB/cm. At a wavelength of 1.31 µm and 1.55 µm, the cross-talk of 462 dB and 107.1 dB was achieved, respectively, using 224 nm and 504 nm bandwidths [28]. Yan et al. published a new gold-plated PCF polarization filter in 2020. There were two big air holes in the filter, which was selectively coated with gold after being filled with water. At 1310 nm, the confinement loss in the Y-polarized mode was 1209.57 dB/cm, but the confinement loss in the X-polarized mode was almost non-existent [29]. Lavanya et al. reported a combined silver–graphene layered pentagonal PCF filter in the year 2021. The filter renders an X-polarized mode with losses of 0.12 dB/cm and 0.59 dB/cm at wavelengths of 1.31 µm and 1.55 µm, respectively. It also rejects a Y-polarized mode with a loss of 361.26 dB/cm and 1508.37 dB/cm, respectively. At 1.31 µm and 1.55 µm, respectively, the cross-talk of 216.68 dB and 904.67 dB was obtained for fiber lengths of 300 µm. [30]. Coating a metal in the inner part of the fiber is complex and hard to fabricate, but it is used in the majority of the filters. A D-shaped plasmonic fiber has been proposed to tackle this problem, with the top circular portion of the fiber removed and the top surface polished. In 2018, Ying et al. reported a D-shaped PCF single-polarization filter. A micro-opening had formed at the top of the PCF, and a gold layer was coated on it. At 1.55 µm, the Y-polarized mode’s confinement loss reached 376.31 dB/cm, while the X-polarized mode's loss was 0.17 dB/cm. For a fiber length of 1 mm, a bandwidth of 480 nm was attained with a crosstalk of 326.7 dB/cm [31]. In 2019, Almewafy et al. proposed an improved D-shaped filter that deposits gold on both sides. At an operating wavelength of 1.3 µm and 1.52 µm, the crosstalk was 46.4 and 41.2 dB, respectively, while the bandwidth was 45 nm and 110 nm for a fiber length of 23 µm [32]. In 2020, Shima et al. proposed a bimetallic coated D-shaped PCF-based single-polarization filter. A Y-polarized mode’s confinement loss reached 950.68 dB/cm at a wavelength of 1.55 µm. The bandwidth was 380 nm, with an extinction ratio of 806.52 dB [33].

SPR technique is also widely used in sensing applications because of its accuracy and extreme sensing performance. At the phase-matching condition, maximal energy transferred from the fundamental mode to the plasmon mode causes a shift in resonance wavelength. The resonance wavelength shift method is used in the SPR-based sensor. It can detect slight variation in the refractive index of the analyte. The external sensing technique is used because the filling and removal of analytes within the structure are complicated [34]. Rifat et al. proposed a PCF-based SPR sensor. For better detection, silver was utilized as a plasmonic material and was coated with a graphene layer. The external sensing technique was used to detect the refractive index of the analyte and obtained the sensitivity of 3000 nm/RIU for the sensing range of 1.46–1.49 [35]. The surface-plasmon-based sensors using silver as a coating material are being reported by many authors in their literature [14,15,36].

In this paper, a new simple reconfigurable surface-plasmon-based filter/sensor using D-shaped photonic crystal fiber is proposed. The proposed structure can be used as a polarization filter without a filling analyte and, at the same time, it can act as a sensor when it is filled with an analyte, which is interested in being sensed. The asymmetrical core design helps enhance the birefringence and, hence, it exhibits the difference in X and Y-polarization propagation of a fundamental mode. This principle helps the fiber realize it as a polarization filter. By using the phase-matching condition between the surface plasmon mode and either one polarization mode of the fiber, particular polarization can be filtered. A tiny layer of silver is placed on the flat surface of the D-shaped fiber with a small half-circular opening to activate the plasmon modes. Various structural characteristics, such as silver thickness, air hole size and shape, and pitch size, are tuned to improve the filter’s filtering efficiency. As the second part of our proposed work, the same structure is extended as a sensor by filling analytes in the outer circle of the proposed filter design. The performances of both polarization filter and RI sensor are analyzed using the finite element method (FEM). The rest of the paper is organized as follows. Section 2 presents the design of the D-shaped PCF. Section 3 discusses the results for the proposed PCF as (Section 3.1) a filter and (Section 3.2) a sensor, and finally, the conclusion is bestowed in Section 4. 

## 2. Design of D-Shaped Photonic Crystal Fiber

**Figure 1:**
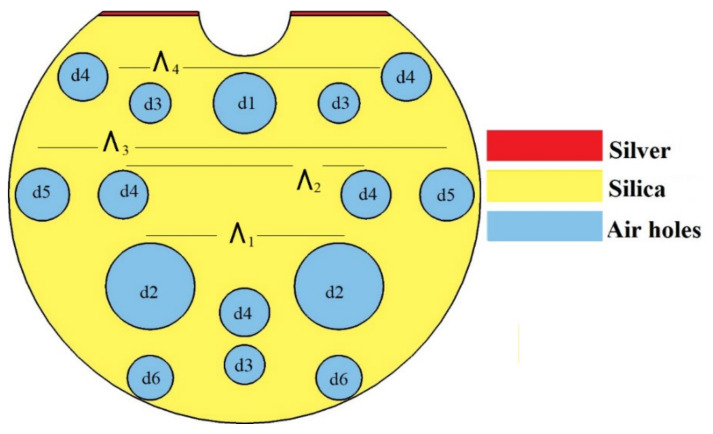
Proposed silver lining D-shaped PCF (for both filter and sensor applications).

Figure 1 shows the schematic diagram of a D-shaped PCF with a silver layer coated on its top surface. In the proposed structure, the air holes are arranged in a hexagonal lattice with 3.5 µm lattice size. The air holes that have different diameters ranging from 1.5 to 3.3 µm form a single-core PCF. Here, a unique PCF structure with minimal number of air holes is used for higher light confinement. Figure 1 illustrates the schematic diagram of the proposed reconfigurable D-shaped PCF filter/sensor. D-shaped PCF is formed by slicing the PCF at 8 μm from the core center. The 15 nm silver layer is coated on the top surface of the PCF, which acts as the noble metal. In the proposed structure, the air holes are arranged in a hexagonal lattice with a 3.5 µm lattice size forming a single-core PCF. All air holes have different diameters, ranging from d1 to d6. The values of these diameters are as follows: d1 = 2.3 μm, d2= 3.3 μm, d3 = 1.5 μm, d4 = 1.84 μm, d5 = 2 μm and d6 = 1.7 μm. The pitch value (Ʌ_1_) between the d2, d3 and d6 air holes is 7 μm. The pitch values Ʌ_2_ = 9 μm, Ʌ_3_ = 15 μm and Ʌ_4_ = 12 μm are maintained between d4, d5 and top d4, respectively. The air holes d1, d2 and d3 are located at 3.5 μm from the center in the Y axis. Air holes d4 are located at 4.5 μm from the center in the Y axis. Air holes d6 and d3 are located in the Y axis from the center at 7 μm and 6.5 μm, respectively. The chosen diameter of the PCF is 17.5 μm. A minimal number of air holes were preferred for higher confinement and also to obtain a unique structure. Here, the missing air holes are used to create an asymmetric core that helps achieve single-mode polarization filtering by increasing the birefringence. A micro-opening is created in the middle of the top side with a diameter of 3.4 μm to influence the phase-matching condition between the core mode and the plasmon mode using analyte. Furthermore, this micro-opening also provides two channels, allowing for improved interaction between the core and plasmon modes.

Because of the short resonance peak when compared to gold and its resonance capability from visible to infrared wavelength range, silver is preferred as the plasmonic material [36,37]. A thin layer of silver with thickness of 15 nm is deposited on these two pathways. SPR takes place through this silver layer. Light propagating through the core induces plasmons to develop in the silver layer. The maximum energy couples from the core mode to the plasmon mode at the phase-matching condition. This coupling creates a decline in energy in the core mode, which attenuates one of the polarized lights while allowing the other due to its birefringence behavior. As a result, the PCF can act as a polarization filter. The scattering boundary condition is established to minimize scattering losses. The performance evaluation of the proposed system is carried out using the finite element method (FEM). The proposed filter structure can be fabricated using the stack-and-draw approach [38] and mechanical side polishing methods [39]. The chemical vapor deposition method is used to deposit the silver lining [40].

Pure silica is used as a core material in this PCF-based Polarization filter, and its refractive index value is calculated using the Sellmeier equation [41]:

(1) $$n{\left(\lambda \right)}^{2}=\frac{A_{1}\lambda^{2}}{\lambda^{2}-\lambda_{1}^{2}}+\frac{A_{2}\lambda^{2}}{\lambda^{2}-\lambda_{2}^{2}}+\frac{A_{3}\lambda^{2}}{\lambda^{2}-\lambda_{3}^{2}}$$

where, A_1_ = 0.6961663, A_2_ = 0.4079426, A_3_ = 0.897479, λ_1_ = 0.068404, λ_2_ = 0.1162414, λ_3_ = 9.896161 are the Sellmeier coefficients.

Dielectric constant value of silver is calculated using the Drude dispersion model [42,43]:

(2) $$\varepsilon_{\mathrm{Ag}}=1-\frac{\lambda^{2}\lambda_{C}}{\lambda_{p}^{2}\left(\lambda_{c}+i\lambda \right)}$$

where collision wavelength λ_c_ = 17.614 μm, Plasma wavelength λ_p_ = 0.14541 μm, and λ being the free space wavelength.

Confinement loss(α) of the filter can be determined numerically using the following equation [44]:

(3) αdB/cm=8⋅686×2πλ×Imneff×104

where Im(n_eff_)—imaginary part of effective mode index.

**Figure 2:**
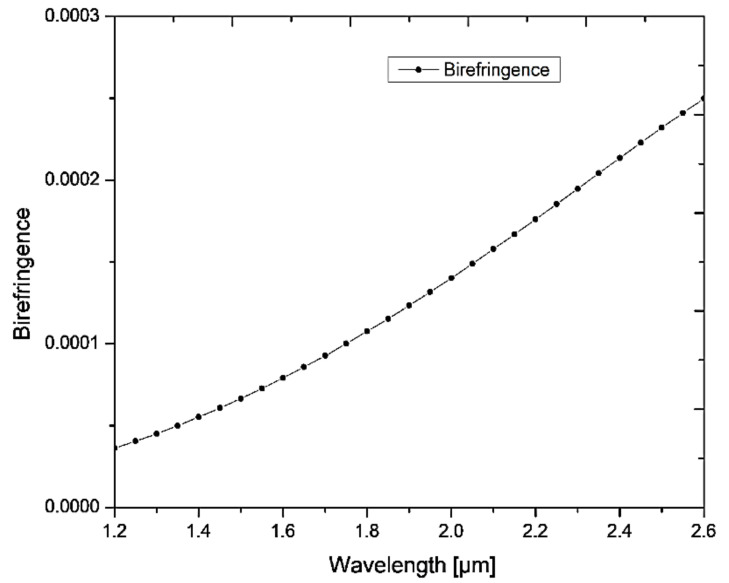
The birefringence vs wavelength of the proposed fiber without silver lining and analyte.

Figure 2 shows the birefringence value of the proposed fiber without silver lining and analyte. The observed birefringence values are 0.73 × 10^−4^, 1.08 × 10^−4^ and 1.76 × 10^−4^ at wavelengths 1.55 μm, 1.8 μm and 2.2 μm, respectively. These birefringence values are of the order of 2 to 3 times more than the normal PCF. This ensures the SPR at different wavelengths for the X and Y-polarization mode. Consequently, this enables the polarization filter function and sensing behavior at a particular polarization using the proposed PCF. 

## 3. Results and Discussion

### 3.1. SPR Based Filter

#### 3.1.1. Optimization of the Structural Parameters

To obtain an optimal filter characteristic, the effects of structural parameters such as size and shape of the air holes, lattice size, and silver layer thickness are examined. By changing one of these parameters iteratively while keeping the others constant, the performance of the proposed PCF filter design is examined. 

#### 3.1.2. Varying Size of Air Holes

**Figure 3:**
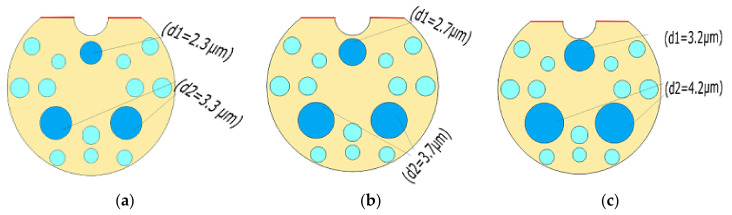
D-shaped PCF based polarization filter with different air hole’s sizes (**a**) air holes dia d1 = 2.3 µm and d2 = 3.3 µm (high lattice) (**b**) air holes dia d1 = 2.7 µm and d2 = 3.7 µm (Mid lattice) (**c**) air holes dia d1 = 3.2 µm and d2 = 4.2 µm (low lattice).

**Figure 4:**
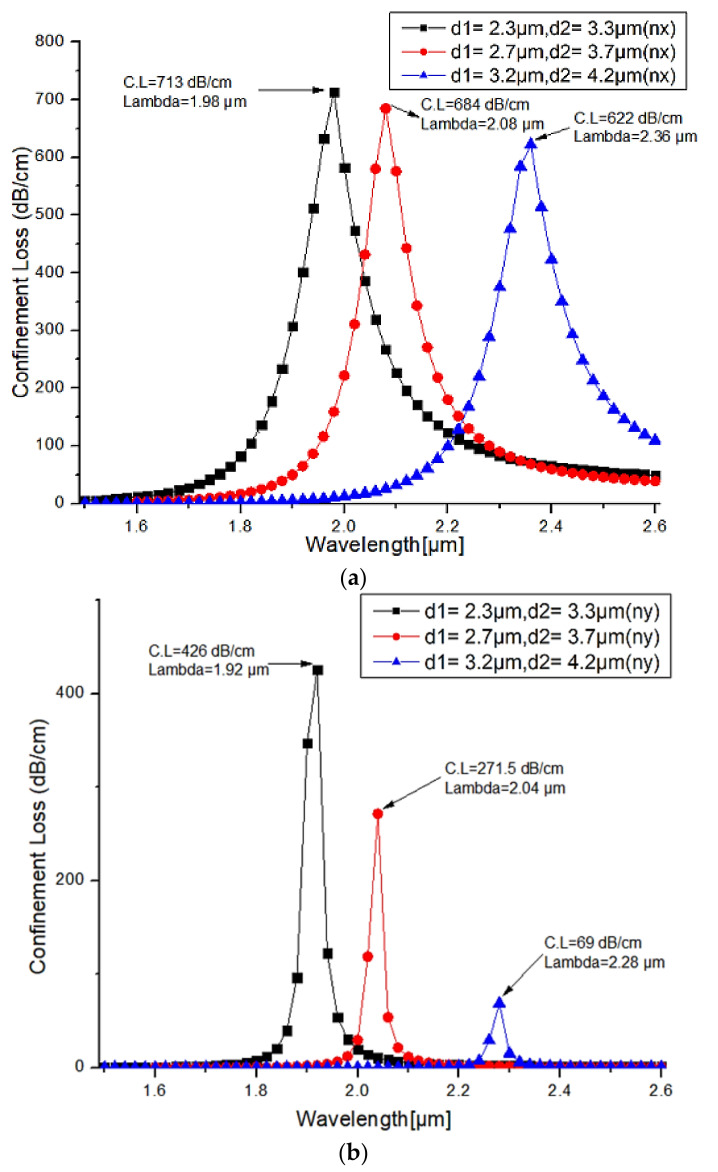
Confinement loss for the different pitch values (**a**) X-polarized mode (**b**) Y-polarized mode.

The filter characteristics of the proposed PCF structure measured for different air holes sizes are displayed in Figure 3. The confinement loss is realized using the surface resonance and it is measured for different air holes diameter values as illustrated in Figure 4a,b for both X-polarization and Y-polarization. As the air holes size decreases (high lattice), the sharp confinement loss peak shifts towards shorter wavelengths. The effective area of the core is increased as the air holes sizes are reduced. At phase-matching conditions, more energy couples from core mode to plasmon mode and it creates more confinement loss at core mode. A confinement loss of 713 dB/cm has been reported at 1.98 µm for X-polarized mode with a pitch of 3.5 µm. For Y-polarization the observed confinement loss is 426 dB/cm at 1.92 µm.

#### 3.1.3. Varying Shape of the Air Holes 

**Figure 5:**
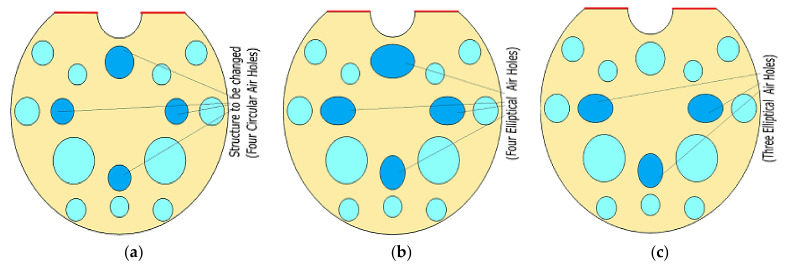
D-shaped PCF-based polarization filter with different air hole structure (**a**) all-circular air holes (**b**) with three elliptical air holes (**c**) with four elliptical air holes.

To find the optimal filter structure, different airhole shapes were chosen, from circular to ones depicted in Figure 5a–c. As the diameter of the air holes increases, the effective area of the core mode decreases, causing the resonance peak to shift toward shorter wavelengths. Figure 5a shows PCF with standard circular air holes. Additionally, the modified three elliptical air holes and four elliptical air holes are shown in Figure 5b,c respectively.

**Figure 6:**
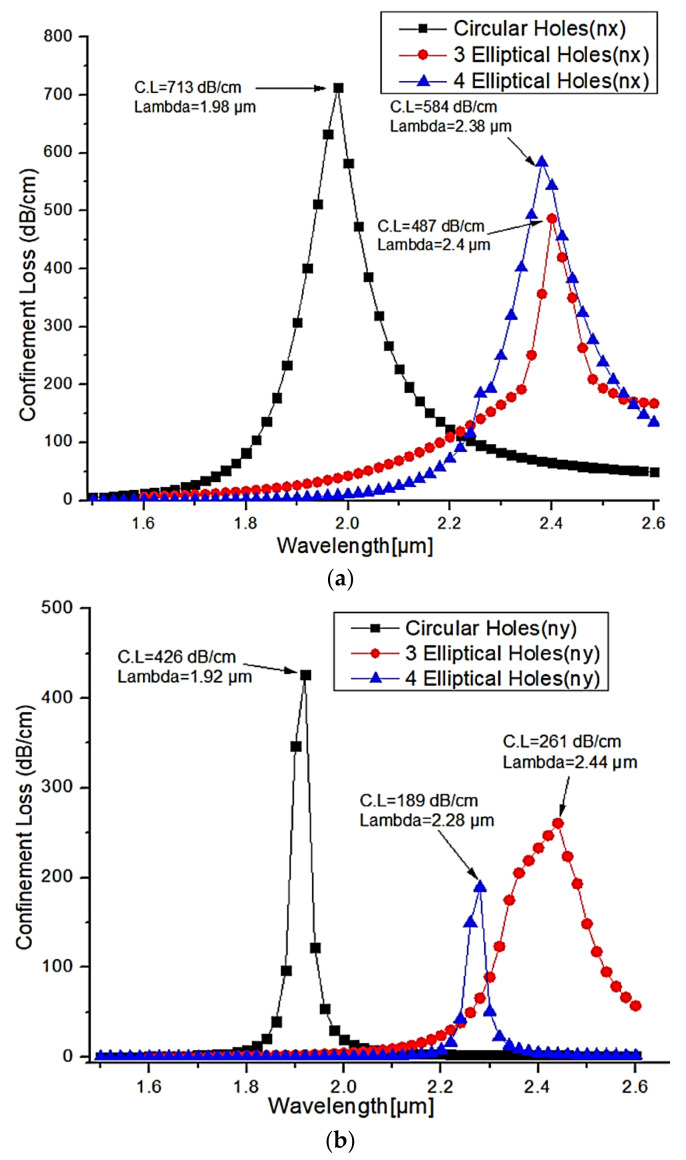
The confinement loss for the different shapes of air holes in (**a**) X-polarized mode (**b**) Y-polarized mode.

Figure 6a,b show the comparison of confinement loss peaks for the X-polarization and Y-polarization with different structures of air holes. As shown in Figure 5, circular air holes, three elliptical air holes and four elliptical air holes are preferred to improve the structure’s efficiency and thereby find the optimal structure. For wavelengths ranging from 1.5 µm to 2.6 µm, numerical analysis was carried out with a step value of 0.02. From this analysis, the observed highest confinement loss for circular air holes is about 713 dB/cm at an operating wavelength of 1.98 µm. For three elliptical holes, the observed confinement loss is 584 dB/cm at the operating wavelength of 2.38 µm. For four elliptical holes, the confinement loss is 487 dB/cm at an operating wavelength of 2.4 µm. From this graph of X-polarization, the circular air holes have the highest confinement loss compared to three elliptical holes and four elliptical holes; the difference is fairly high. The circular air holes are only carried for further analysis due to their superior performance in terms of their confinement loss. 

#### 3.1.4. Varying the Thickness of Silver

**Figure 7:**
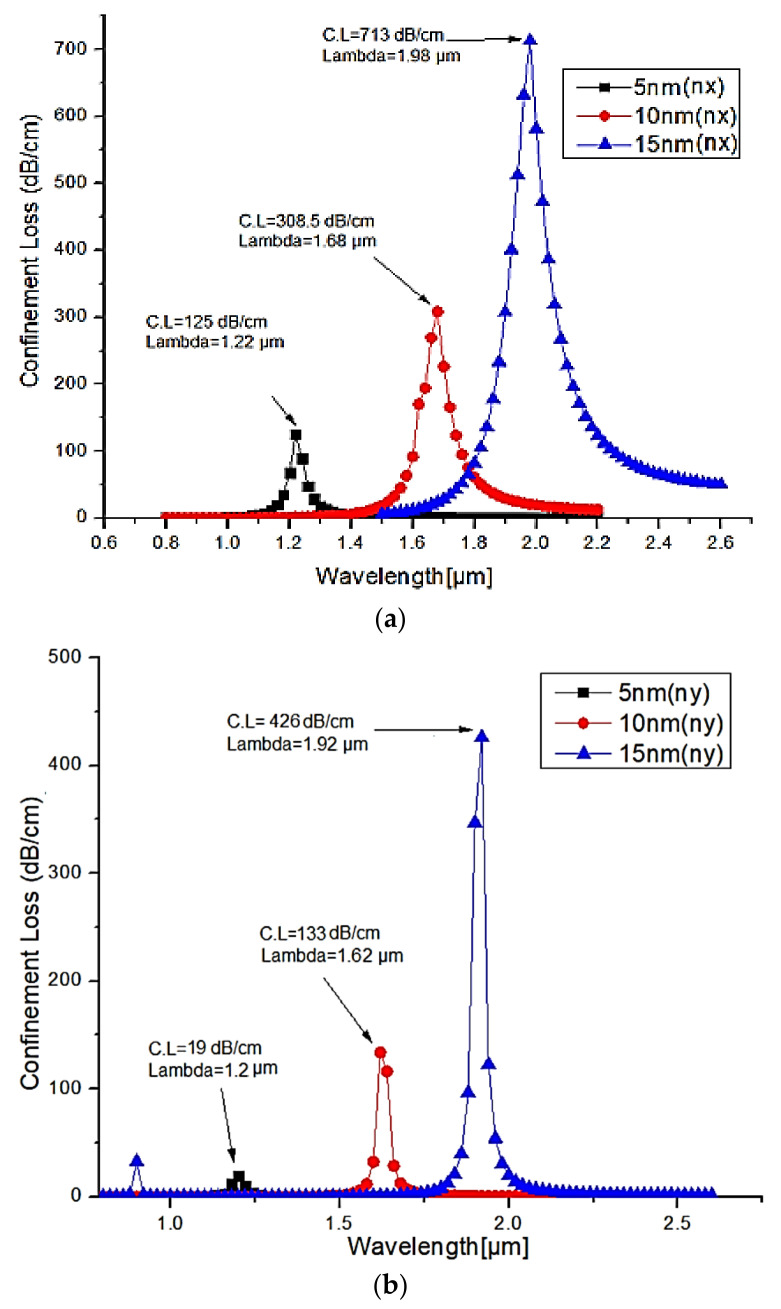
Characteristics of confinement loss for various silver layer thicknesses (5, 10 and 15 nm) (**a**) X-polarized mode (**b**) Y-polarized mode.

The silver lining thickness has an impact on the efficiency of the plasmonic based PCF filter by changing the plasmonic resonance. As per Chao Liu, et.al. [45], 20 nm thickness of silver provides a better sensitivity among the thickness range from 20 to 50 nm. It clearly states that less thickness provides a better surface plasmon resonance compared to the higher thickness level and hence it is decided to carry out further research to improve the sensitivity of the sensor by changing the silver coating thickness in the range between 5 nm and 15 nm with a step size of 5 nm. Out of this research, it is found and reported here that 15 nm thickness of Ag provides good confinement loss and wavelength sensitivity as shown in Figure 7a,b. Under phase matching conditions, the observed maximum loss is 713 dB/cm for the X-polarized mode at the operating wavelength of 1.98 µm. Also, the maximum loss of 426 dB/cm is recorded at the operating wavelength of 1.92 µm for Y-polarized mode.

#### 3.1.5. Transmission Characteristics and Dispersion Relation

**Figure 8:**
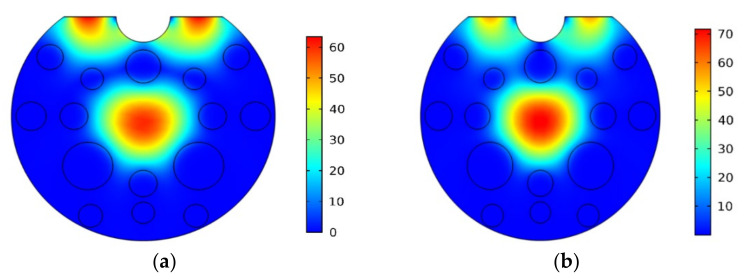
Modal field distribution (**a**) Resonance condition for X-polarization at 1.98 μm (**b**) Resonance condition for Y-polarization at 1.92 μm.

For the optimized structure of the filter, the light coupling characteristics are investigated over a wavelength range from 0.8 to 2.2 µm in both X and Y-polarizations. The maximum energy of the core mode is transferred to SPP mode at the phase-matching condition, and the energy of the core mode rapidly decreases. As a result, it will attenuate one of the polarized lights while allowing the other to go through. Figure 8a,b illustrate the electric field distributions of X-polarized mode at wavelength 1.98 μm and Y-polarized mode at wavelength 1.92 μm.

**Figure 9:**
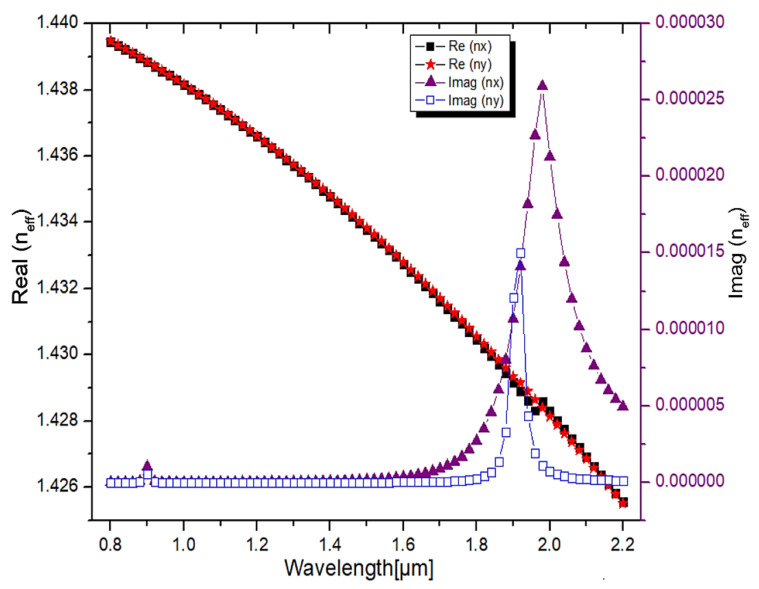
Real and imaginary parts of the PCF filter core guided mode as a function of wavelength.

The dispersion relation between the real and imaginary values of the effective mode index (n_eff_) is depicted in Figure 9. As shown in Figure 9, the real part of the core-guided mode decreases when the wavelength increases. The fiber dispersion characteristic is defined by the real values of n_eff_ [46], and the confinement loss is calculated through imaginary values of n_eff_ [47]. As depicted in Figure 9, the X-polarized mode couples with the plasmon mode, and hence, the core mode imaginary RI value increases at 1.98 µm. The observed confinement loss is 713 dB/cm at this wavelength, whereas the Y-polarized mode couples with the plasmon mode and has a confinement loss of 426 dB/cm at the wavelength of 1.92 µm. As per the result, at the wavelength of 1.98 µm, only the Y-polarized mode propagates, and the X-polarized mode is attenuated, and at the wavelength of 1.92 µm, only the X-polarized mode propagates, and the Y-polarized mode is attenuated. As a result, the proposed structure functions as a polarization filter by removing one of the polarized light waves at a particular wavelength.

### 3.2. SPR Based Sensor

**Figure 10:**
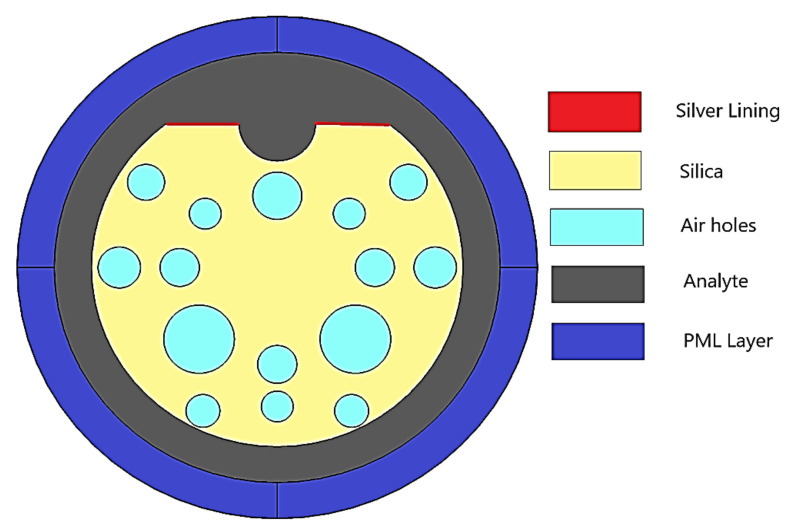
Graphical representation of the SPR based sensor structure.

The optimized SPR-based polarization filter structure extends to sensing application by filling an analyte in the outer layer of the sensor. When the refractive index of the analyte changes, the structure resonant wavelength will shift. For the sensing purpose, the resonance wavelength shift (wavelength sensitivity) scheme is preferred because it results in a high level of sensitivity [48]. A schematic representation of the plasmonic sensor is shown in Figure 10. A PML layer is used to reduce scattering losses. The analyte’s indices influence the amount of light coupled into the silver layer (surface plasmon mode) and thus deciding light propagation through the core. 

**Figure 11:**
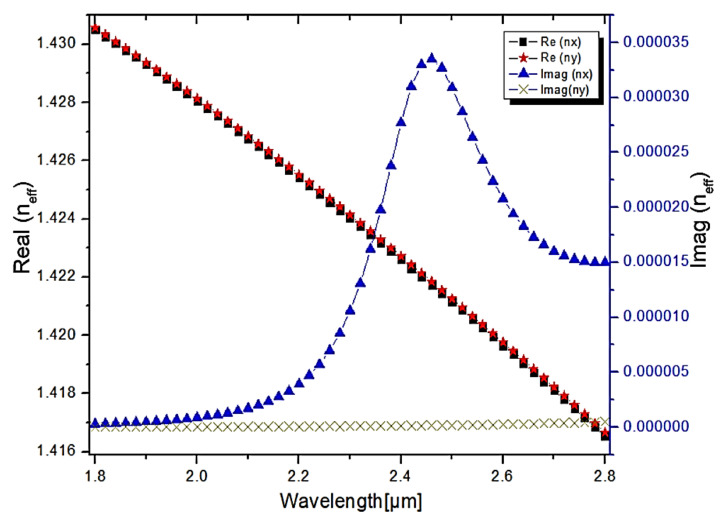
Real and Imaginary values of 1.36 RI.

Figure 11 depicts the real and imaginary values of the core-guided mode for the analyte RI of 1.36. As shown in Figure 11, the real part of the core-guided mode decreases as the wavelength increases. The fiber’s core-guided mode is coupled with the plasmon mode at a wavelength of 2.46 µm. As a result, maximum energy is coupled from the core mode to the plasmon mode, causing fundamental mode loss. At the resonance condition, the imaginary value of the core-guided mode reaches its maximum value. The performance characteristic of a sensor is defined by its sensitivity, and thus, sensitivity needs to be analyzed. Sensitivity (wavelength shift) of a sensor is calculated [49] as follows,

(4) $$S_{\lambda }=\frac{{\Delta \lambda }_{\mathrm{Peak}}}{{\Delta n}_{a}}\left(\mathrm{nm}∕\mathrm{RIU}\right)$$

Δ𝜆_peak_–resonance wavelength shift, Δn_a_—analyte RI value shift.

**Figure 12:**
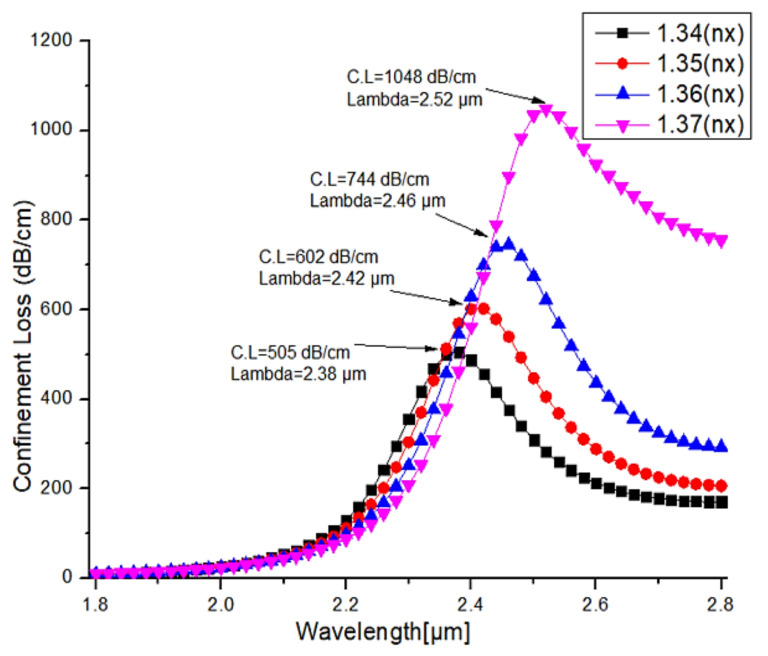
Confinement losses in both X and Y-polarizations for the different RI ranging from 1.34 to 1.37 with step value of 0.01.

**Figure 13:**
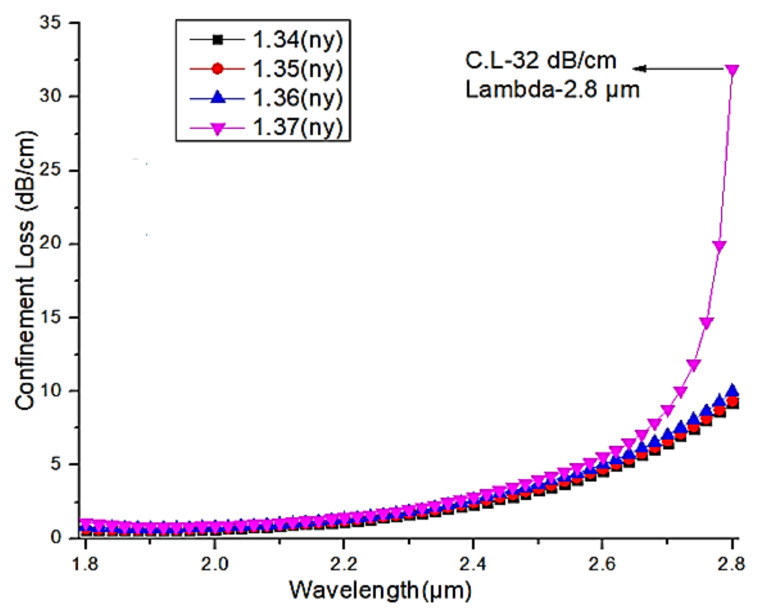
Confinement loss in Y-polarization with respect to wavelength (extracted from Figure 12 for more clarity).

For the proposed sensor, a numerical investigation was carried out over a wavelength ranging from 1.8 µm to 2.8 µm, with a step value of about 0.02 µm. Additionally, for the analytes, the refractive indices ranging from 1.34 to 1.37 in the step of 0.01 were taken for the analysis. Figure 12 and Figure 13 illustrate the confinement loss measured in dB/cm for both the X and Y-polarization. As shown in Figure 12, the observed confinement losses at the X-polarization are 505 dB/cm, 602 dB/cm, 744 dB/cm, 1048 dB/cm at wavelengths of 2.38 µm, 2.42 µm, 2.46 µm, 2.52 µm, for the analyte refractive indices of 1.34, 1.35, 1.36, 1.37, respectively. From this analysis, the maximum confinement loss of about 1048 dB/cm is observed for the analyte refractive index of 1.37 at the operating wavelength of 2.52 µm, whereas in the Y-polarization, the observed confinement losses are very low, as depicted in Figure 12, and for the refractive indices of 1.34, 1.35, 1.36, 1.37, the recorded maximum values at the wavelength of 2.8 µm are 9.2 dB/cm, 9.5 dB/cm, 10 dB/cm, 32 dB/cm, respectively. Figure 13 is an extracted image from Figure 12 to display the confinement loss at the Y-polarization. From this analysis, it is clearly shown that the proposed PCF structure can be used as a refractive index sensor by shifting the confinement loss with respect to the wavelength in the X-polarization for the different analyte indices. 

**Figure 14:**
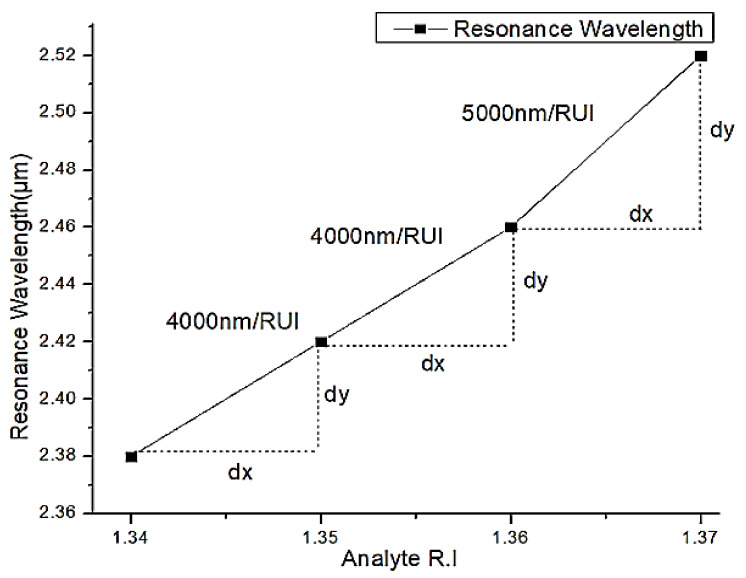
Sensor sensitivity measurement using resonance wavelength shift with reference to analyte RI changes.

The sensitivity of the D-shaped silver lining plasmonic sensor is calculated using Equation (4). The sensitivity of the proposed system over the RI ranging from 1.34 to 1.37 is depicted in Figure 14. The observed sensitivity from RI 1.34 to 1.36 is 4000 nm/RIU, and the sensitivity is improved to 5000 nm/RIU for the RI ranging from 1.36 to 1.37. This sensor can be implemented to detect the small changes in the bio analytes and industry chemicals. The comparison of the proposed work with contemporary literature works is as depicted in Table 1. It shows a good wavelength sensitivity in the proposed work over the RI range from 1.34 to 1.37. This RI range covers the sensing of many industrial solvents and bio-analytes [50] RIs.

Compared to the literature works [10,13,15,51,55,59], the proposed work provides superior wavelength sensitivity of 5000 nm/RIU, approximately, for the RI ranges from 1.33 to 1.40. The reported work in Ref. [52] covered exactly the RI range of 1.34–1.37, similar to this proposed work, with a sensitivity of about 9000 nm/RIU. However, the PCF reported in Refs. [10,52] had the air hole at the axis of the fiber, with a dual channel on either side of the fiber axis to sense the change in RI values. Compared to these literature works, the proposed work is very simple in design and less complex to couple the light for the polarization-sensitive sensors. Contrarily, the literature works [10,52] will create losses while coupling the light from an external single-mode fiber due to the presence of the airhole at the center. Moreover, this air hole at the fiber axis will create complexity to couple the light with proper polarization in polarization-sensitive sensors.

**Figure 15:**
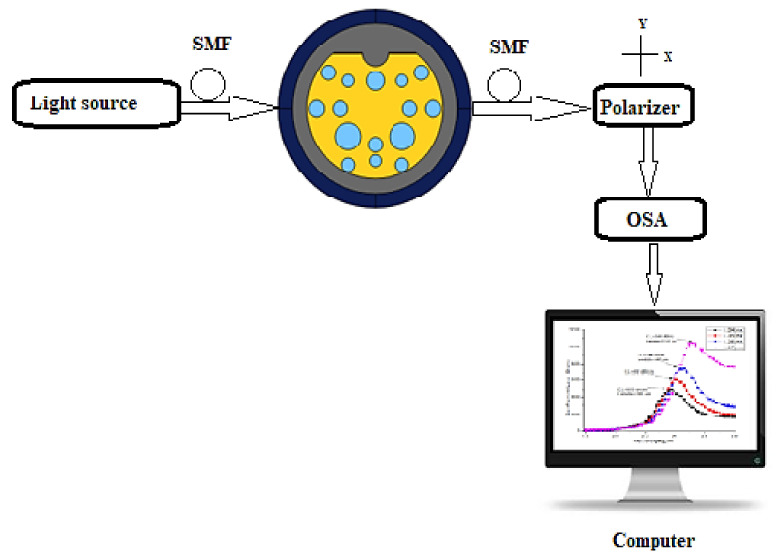
Experimental arrangement for the proposed reconfigurable D-shaped SPR filter/sensor.

Figure 15 depicts the experimental arrangement for the proposed reconfigurable D-shaped SPR filter/sensor. A light source with a wide spectrum is launched into the proposed fiber through SMF for better coupling. Depending on the analyte being filled or unfilled around the proposed fiber, this setup can act as a polarization filter or sensor. As a sensor, the addition of a polarizer gives better visibility regarding the confinement loss under a particular polarization. As per the RI of the analyte, the fiber mode loss varies at a particular wavelength, and it can be recorded on an optical spectrum analyzer (OSA) and computer connected to it. By analyzing the data from the computer, the unknown analyte RI can be identified through the wavelength shift of peak loss in SPR between the core mode and the plasmon mode.

## 4. Conclusions

SPR based silver lining reconfigurable D-shaped photonic crystal fiber is proposed to filter out the single-polarization and to sense the RI of analytes. A tiny layer of silver is deposited over the flat surface of the D-shaped fiber with a small half-circular opening to activate the plasmon modes. Structural optimization has been done through FEM to obtain a single polarization filter and the performance parameters are measured. For the application of PCF as a filter without adding analyte, the maximum confinement loss of 713 dB/cm is observed in X-polarization at the wavelength of 1.98 µm and for Y-polarization the maximum confinement loss is observed as 426 dB/cm at 1.92 µm. This property allows the proposed structure to act as X or Y-polarization filter at the wavelength of 1.98 µm and 1.92 µm respectively. This PCF filter can be used as a sensor by adding an analyte outside this filter structure. With a maximum sensitivity of 5000 nm/RIU at X-polarization, this sensor can detect small refractive index (RI) variation of analytes ranging from 1.34 to 1.37. This sensor can be used for industrial and medical applications to measure chemical concentrations and disease levels using sample analytes. In future, research can be focused towards finding a good improvement in the dynamic range of sensing properties by extending the same structure with different materials like chalcogenide, Fluoride glass and Bismuth oxide instead of silica.

## Figures and Tables

**Table 1:** Comparison of our proposed work with contemporary literatures.

Year/Ref	Plasmonic Material Used	Application Demonstrated	Sensitivity [nm/RIU]	RI Range
2015/[37]	Silver/Graphene	Sensor	3000	1.46–1.49
2017/[51]	Gold	Sensor	4000	1.33–1.37
2018/[10]	Gold	Sensor	4600	1.33–1.38
2018/[52]	Gold	sensor	9000	1.34–1.37
2019/[15]	Silver/Graphene	Sensor	3750	1.33–1.35
2019/[53]	Silver/Graphene	Sensor	833.33	1.30–1.34
2020/[13]	Silver/Gold	Sensor	3083	1.33–1.366
2020/[54]	Gold/Titanium dioxide	Sensor	4782	1.49–1.54
2021/[55]	Gold/polydimethylsiloxane	Sensor	1371	1.33–1.34
2022/[56]	Silver	Sensor	4100	1.38–1.41
2022/[57]	Silver	Sensor	1932	1.25–1.30
2022/[58]	Gold	Sensor	3000	1.40–1.46
2022/[59]	Gold	Sensor	3300	1.35–1.40
Proposed work	Silver	Filter/sensor	5000	1.34–1.37

## Data Availability

Not applicable.
